# Development of an Evidence-Based Cognitive Training Application for Elderly Individuals with Cognitive Dysfunction

**DOI:** 10.3390/healthcare13030215

**Published:** 2025-01-21

**Authors:** Hee-Jae Chae, Chan-Hee Kim, Seon-Heui Lee

**Affiliations:** 1 Department of Nursing Science, College of Nursing, Gachon University, Incheon 21936, Republic of Korea; chae951021@gachon.ac.kr; 2 Department of Nursing, Graduate School, Yonsei University, Seoul 03722, Republic of Korea; potbeau@gmail.com; 3 College of Nursing, Research Institute of AI and Nursing Science, Gachon University, Incheon 21936, Republic of Korea

**Keywords:** cognitive dysfunction, community health nursing, dementia, mobile applications, information technology

## Abstract

Background: Early cognitive training is important to prevent cognitive decline in patients with mild cognitive impairment (MCI) or dementia. Therefore, developing an application that can provide evidence-based cognitive training is necessary for patients with MCI or dementia. Method: This study aimed to develop and evaluate Smart Brain, an evidence-based application that provides comprehensive cognitive training tailored to this population. The application was developed using an ADDIE (analysis, design, development, implementation, and evaluation) model. A systematic review of databases, including Ovid-MEDLINE, Ovid-EMBASE, Cochrane Library, and CINAHL, was conducted up to April 15, 2021, to identify key content areas. Additionally, a survey of 100 participants highlighted the need for features such as cognitive games, health notes, social networking services, and goal achievement. Result: The application was developed with distinct user and administrator interfaces to support engagement and monitoring. Usability testing involved 7 experts and 11 elderly individuals with MCI or dementia from a daycare center. Based on usability feedback, features such as the time limits for cognitive games were refined. The final application integrates cognitive games, physical exercises, emotional support, and health management tools to address user needs comprehensively. Conclusion: Smart Brain holds significant potential to improve the quality of life and cognitive health of elderly individuals with MCI or dementia. Its usability and functionality make it a promising tool for community-based interventions.

## 1. Introduction

Dementia is a syndrome characterized by difficulties with memory, language, problem-solving, and other cognitive skills [1]. It involves objective cognitive decline with repercussions on the individual’s previous functional autonomy. As the proportion of elderly individuals is increasing in a majority of countries, the number of elderly people with dementia is also increasing. The cost of caring for elderly individuals with dementia is expected to exceed USD 2.8 trillion by the year 2030 [2]. Mild cognitive impairment (MCI) is classified as an intermediate between normal cognitive function and dementia. MCI is recognized as a risk factor for developing dementia, though not all individuals with MCI progress to dementia [3]. Key predictors for dementia progression include older age, lower cognitive function, and impaired social relationships [3]. Identifying and addressing these predictors through early interventions may help delay or prevent the onset of dementia, improving outcomes for both patients and caregivers [4,5].

A variety of pharmacological and non-pharmacological interventions can slow the progression of dementia and improve the well-being of patients and their caregivers [6,7]. Recent pharmacological advancements, such as disease-modifying treatments for Alzheimer’s disease like Lecanemab and Donanemab, have demonstrated efficacy in altering the clinical and biological course of the disease [8,9]. Among non-pharmacological treatments, FINGER-type multimodal interventions, which combine exercise, cognitive training, dietary improvements, and vascular risk management, have shown the strongest evidence for improving cognitive and functional outcomes in individuals at risk of cognitive decline [10]. Other non-pharmacological approaches, such as music therapy, cognitive stimulation, exercise, psychological treatment, and digital treatment [11], have also been widely utilized to support cognitive and emotional well-being.

Building on these holistic approaches, the development of information and communication technology (ICT) has expanded the potential for delivering multimodal interventions more efficiently and at scale. The need for such innovations became particularly evident during the COVID-19 pandemic, when many individuals, including elderly individuals with dementia, required remote support services to maintain independence and functionality [12]. ICT-based interventions utilize artificial intelligence (AI), wearable technology, and the Internet of Things (IoT) to monitor cognitive performance and provide real-time feedback. For example, wearable devices collect biosignals such as heart rate variability and sleep patterns [13], while AI-driven analytics personalize interventions to meet individual needs [14]. These interventions have demonstrated promising results in improving cognitive functions such as attention [15], memory [16], and language abilities [17] in elderly individuals with MCI or dementia [13], while also showing potential benefits in enhancing quality of life and alleviating caregiver burden [18]. Furthermore, ICT-based cognitive intervention has also been effective in improving quality of life [19], physical activity [20], and depression [21]. Therefore, it is necessary to develop cognitive interventions, for elderly individuals with MCI or dementia, using ICT.

Despite these promising results, existing ICT-based cognitive training applications face some limitations. For instance, many programs are narrowly focused on cognitive games, which may fail to engage users in the long term. Additionally, these programs often lack diverse content that incorporates activities such as arts, physical exercises, literature-based activities, and social interactions, which are commonly found in conventional dementia prevention programs [22]. This narrow focus limits the potential of ICT to address the holistic needs of elderly individuals with MCI or dementia. It also reduces user engagement and adherence, which diminishes the long-term effectiveness of such interventions. Another challenge is the lack of adaptability and personalization in many ICT applications, which often focus on specific cognitive tasks without considering individual user needs [23]. This can lead to cognitive overload, where overly complex or poorly designed tasks overwhelm users, reducing their participation and the effectiveness of the interventions [24]. Additionally, external factors, such as limited technological literacy among elderly users, further highlight the need for more user-friendly and flexible designs [24].

As a response to these challenges, this study aims to develop comprehensive ICT-based cognitive training programs. The development process incorporates a systematic review, needs analysis, and preference analysis tailored to community-dwelling elderly individuals with MCI or dementia.

## 2. Materials and Methods

### 2.1. Ethics Statement

This study was approved by the International Review Board (IRB) of Gachon University (IRB no. 1044396-202101-HR-019-02). Additional measures were taken to safeguard user data. Personal information, such as names and health-related data, was anonymized and securely stored. Identifiable data will be destroyed after the study, while anonymized data will be retained for five years on a secure platform. Participants have the right to request data deletion at any time, and confidentiality will be strictly maintained throughout the study.

### 2.2. Study Design

**Figure 1:**
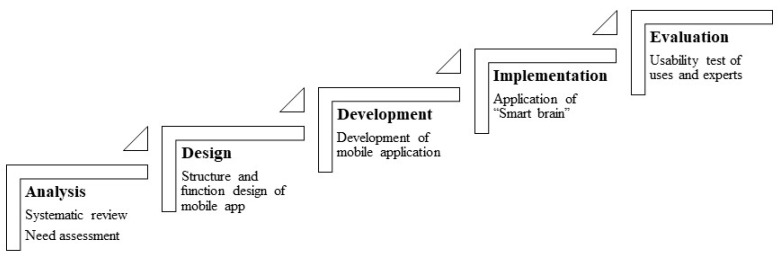
Development process of mobile application using ADDIE model.

This study aimed to develop cognitive interventions using a mobile application for community-dwelling elderly with MCI or dementia. We developed the application following the ADDIE (analysis, design, development, implementation, and evaluation) model (Figure 1), which was considered effective in the development of training programs [25]. This model consists of 5 phases as follows: (a) analysis, (b) design, (c) development, (d) implementation, and (e) evaluation phase.

#### 2.2.1. Phase of Development: Analysis Phase

During this analysis phase, it is necessary to conduct a needs assessment and analyze the characteristics of the subject or program [26]. We conducted a systematic review and preference survey in order to construct the contents of the application.

Through the systematic review [6], we examined the duration of the intervention, time per session, and type of domain and their effects on elderly with MCI and dementia. We conducted searches across four databases published up to April 15, 2021: Ovid-MEDLINE, Ovid-EMBASE, Cochrane Library, and CINAHL. We searched for articles using Medical Subject Headings (MeSH) and keywords related to patients and interventions. The search term was divided into two main components: (1) ICT-based interventions, with keywords such as “digital health”, “mobile application”, and “telehealth”; and (2) dementia, using keywords like “dementia”, “cognitive impairment”, and “Alzheimer’s disease”. Details of the comprehensive search strategy and additional keywords can be found in our systematic review [6]. The inclusion criteria were as follows: (a) elderly with MCI or dementia; (b) ICT-based cognitive training; (c) reported on outcomes such as cognitive function, physical ability, psychosocial status, and quality of life; and (d) randomized controlled trials. We excluded animal studies, studies not published in Korean or English, abstracts, conference posters, and those without proper outcomes. Two authors independently screened the titles and abstracts and selected the appropriate studies. Thereafter, complete texts were screened, and appropriate studies were selected.

In addition, a demand and preference survey was conducted to reflect the user’s needs in the program components. Based on the result of the systematic review, we developed a questionnaire for inspecting the needs and preferences of older adults about ICT-based non-face-to-face services. The questionnaire consists of three categories: (a) general characteristics of the survey participants, (b) needs of ICT-based non-face-to-face service, and (c) preferences for proposed service components. The needs assessment survey included five categories: experience using smart devices, the necessity of non-face-to-face services, intention to use such services, the preferred frequency of interventions, and the preferred duration per session. The preference survey comprised 14 items across five categories: cognitive improvement, health care, emotional support, motivation, and caregiver calls. While the needs assessment employed various response formats tailored to each question, the preference survey utilized a 5-point Likert scale. The content validity of the questionnaire was evaluated by three geriatrics professors. A needs analysis of 100 healthy individuals with spouses with dementia was conducted from 15 July 2021, to 31 August 2021. The research assistant explained each question individually to make it easier for the elderly people to answer. 

#### 2.2.2. Phase of Development: Design Phase

In the design phase, the framework and functions of the mobile application were determined based on the contents identified in the analysis stage [27]. The interface was also designed in this stage. We designed user and administrator servers separately. The user server provides comprehensive care for elderly individuals with MCI or dementia. The administrator server can check all the users’ server usage data and can communicate with them.

**Figure 2:**
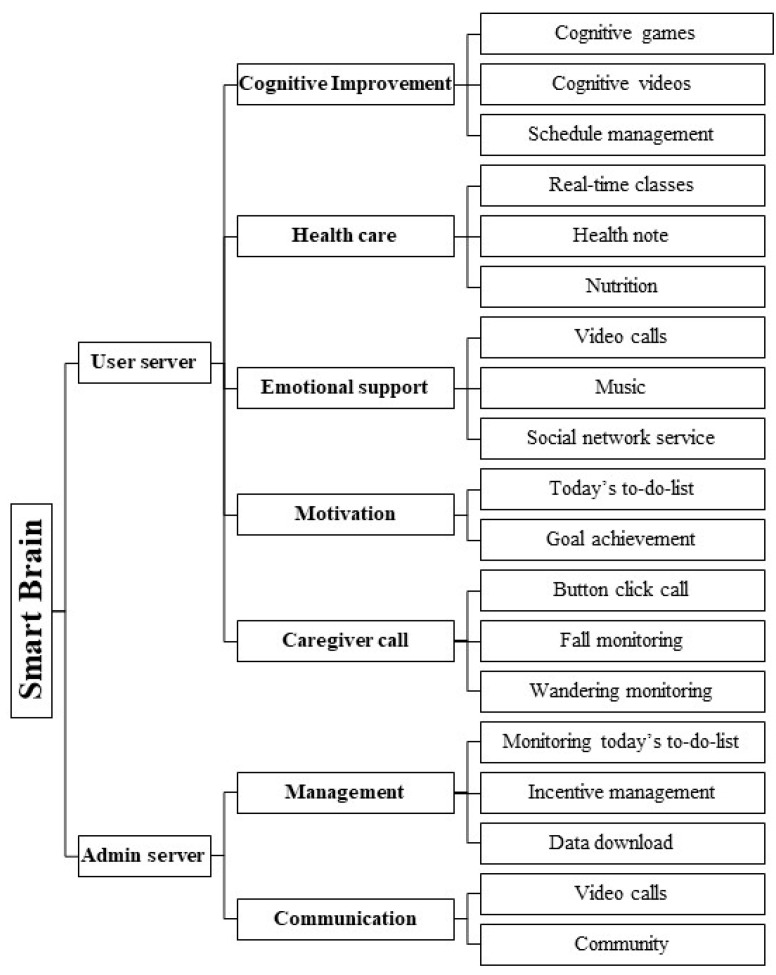
Information architecture of user and administrator server.

The user server’s main menu collects important interventions for easy access. This main menu consisted of five parts: cognitive improvement, health care, emotional support, motivation, and caregiver call based on the results of systematic review and need assessment (Figure 2).

The cognitive improvement component includes cognitive games, cognitive videos, and schedule management. The health care component consists of real-time online classes, health notes, and cognitive improvement nutrition. Emotional support includes video calls, music to listen to, and social networking services. Motivation consists of daily to-do lists and goal achievements. Lastly, the caregiver has click button with call, fall, and wandering monitoring functions. The interface includes an easy-to-read layout, large text, and intuitive pictures for seniors.

We designed an administrator server to provide comprehensive care to the users. The functions of the administrator server are divided into management and communication. In the management function, the administrator could can identify whether a day’s to-do list has been performed, manage the incentive, and access all other data available for download. The interface had intuitive menu names, brief user information, and maximum data utilization.

#### 2.2.3. Phase of Development: Development Phase

During the development phase of generating the materials [25], we collaborated with three companies that included design experts and programmers to create a mobile application. We developed two versions of the application using Java for the Android operating system, one for users and one for administrators, which could be run on a tablet. Furthermore, we established a data transmission method for smart machines, user servers, and administrator servers.

On the user server, various sounds and visual materials were used to stimulate the interest of the elderly with MCI or dementia. The instructional video material was filmed based on a scenario written by an elderly exercise expert or an existing educational video for which permission had been obtained from the author. Furthermore, various sound effects were inserted for when the button was pressed.

The administrator server is convenient for remotely managing users. Records such as access time, time used, and user results were stored on the company’s server, and some could be downloaded by the administrators directly from the server. After the researchers confirmed the completed prototype, the developers and designers made modifications and additions.

#### 2.2.4. Phase of Development: Implementation Phase

The implementation phase was concerned with how the actual application was to be utilized [25]. In this step, seven experts and eleven elderly people were instructed to use the mobile application, which was to be evaluated for usability in the next stage. The seven experts consisted of two geriatric experts, two computational experts, one nursing professor, and two nurses. The application was installed on the tablet by an expert and confirmed by the researchers. Eleven seniors with MCI or dementia attending daycare centers participated in the usability test. 

Usability testing was conducted on a tablet device running Android 9, with a 10.1-inch 1920 × 1200 FHD display, 3 GB of RAM, and 32 GB of internal storage. The device was equipped with a 7000 mAh battery, multiple sensors including a G-sensor, light sensor, hall sensor, and gyroscope, as well as a microphone array with three microphones for voice recognition. 

#### 2.2.5. Phase of Development: Evaluation Phase

This stage refers to the process of evaluating the appropriateness of the developed program [26]. After one week of use, seven experts evaluated the data using the Mobile Application Rating Scale (MARS) [28]. The MARS for experts consists of 23 questions in five sub-areas: engagement, functionality, esthetics, information, and subjective quality. Similarly, the Mobile Application Rating Scale: User Version (uMARS) was used to evaluate 11 users. Similarly to MARS, uMARS consists of five sub-areas, but the “information” area consists of four items, making a total of 20 items. Each item is evaluated on a 5-point Likert scale, and the higher the score, the better the application usability. The Mobile Application Rating Scale (MARS) and its user-adapted version (uMARS) were chosen for this study based on their robust design and established reliability in evaluating mobile health applications. These tools, developed through comprehensive literature review and iterative expert panel refinement, have demonstrated high internal consistency, with a Cronbach’s alpha of 0.90 [28], ensuring their suitability for quality assessment.

Participants for the usability test, which was conducted using uMARS, were recruited from a daycare center in Namdong District. The sample included 11 individuals registered with the center as having mild cognitive impairment (MCI) or mild dementia. Participants were selected based on their ability to provide informed consent and to participate without significant hearing impairments. Evaluations were conducted with the assistance of daycare center staff and researchers, who provided guidance during the testing process. While detailed demographic and clinical characteristics were not collected, all participants voluntarily agreed to participate in the study. 

### 2.3. Statistical Analysis

We analyzed the needs survey and usability test using the SPSS Statistics for Windows (version 26.0; IBM Corp, Armonk, NY, USA) to calculate the mean and standard deviation (SD) for the quantitative variables and the frequency and percentage for the categorical variable. In this study, categorical variables included gender, subjective economic status, education level, and primary caregiver, as listed in the baseline characteristics table. Quantitative variables included age, as well as survey scores from the preference survey and usability assessment.

## 3. Results

In this study, we analyzed the data according to the ADDIE model, which reviews the development process for appropriate goals [29], including the analysis, design, development, implementation, and evaluation phases.

### 3.1. Analysis Phase Findings: Systematic Review and Needs of Spouses of Dementia Patients

Forty-four studies were selected and analyzed from the systematic review [6]. To prevent cognitive decline in the elderly with MCI or dementia, cognitive training such as cognitive games, exercise, education, and the use of wearable devices was conducted. Thirty-seven studies included cognitive programs with or without motivation, four contained health care programs, four included emotional support, and four studies conducted caregiver calls programs. According to the systematic review, cognitive training was effective in improving cognition, depression, and quality of life in elderly individuals with MCI or dementia [6].

From a systematic review that included 44 studies, cognitive training using ICT was shown to have a positive effect on cognitive function, depression, and quality of life in older adults with MCI or mild dementia. Specifically, randomized controlled trials (RCTs) included in the review demonstrated that ICT-based interventions significantly improved Mini-Mental State Examination (MMSE) scores (*p* < 0.05) and reduced depression scores (*p* < 0.05) compared with control groups. The interventions were most effective when they lasted more than 6 weeks, with sessions exceeding 30 min, and incorporated multi-domain approaches. These findings supported the development of the Smart Brain application to provide effective, accessible, and evidence-based cognitive training for community-dwelling older adults. The systematic review, published as a separate study, provides detailed information on the study [6].

The validity of the average item-level content validity for the questionnaire items was 0.99 and importance was 1.00 as given by three professors of geriatric nursing. The spouses of 100 elderly people with dementia were surveyed from June 15 to August 31, 2021. The average age of the survey participants was 73.2 ± 7.0, and the number of female participants was 67 (67%) (Table 1). Regarding subjective economic status, 20 (20%) participants thought that the economic level was low, 67 (67%) thought the economic level was medium, and 13 (13%) thought that it was high. Those with a final qualification of less than or equal to junior high school comprised 37%, and 63% of the participants had a qualification of a high school graduate or higher. Of the respondents, 77 participants (77%) (“strongly need it”, “need it”, and “need it a little”) considered that an ICT -based cognitive training program was needed for elderly individuals in the community who had dementia. However, of the 28 participants who indicated that they would not use the ICT-based cognitive training program, 12 people (42.9%) cited difficulty in learning to operate new devices as their reason for non-participation.

Based on the needs assessment survey, it was found that 47.0% of respondents preferred cognitive stimulation activities lasting less than 30 min per session, and 44.0% suggested sessions between 30 min and 1 h. Since very few respondents (9.0%) indicated a preference for sessions longer than 1 h, the session duration was reduced to less than 1 h, specifically to 30 min, to better align with user preferences. Additionally, missions were considered successfully completed when participants performed assigned tasks three times a week for at least 30 min. These adjustments were informed by both the user needs survey and expert consultations. Furthermore, to address the concern raised by 42.9% of respondents regarding the difficulty of learning to use new devices, we incorporated features such as large fonts, an intuitive interface, and pictograms to enhance usability for older adults. These survey findings directly informed the development of the application’s features, ensuring alignment with user needs and preferences.

In Table 2, regarding the evaluation of the preference for cognitive improvement, “cognitive game” (3.68) was selected as the item with the highest preference. The highest preference in health care was “health note” (3.67). According to the preference survey evaluated by the spouses of dementia patients, social networking services (3.41) had the highest level of emotional care. In terms of motivation, the two were similar, but “goal achievements” was slightly higher at (3.49), and among caregiver calls, “button click call” (3.38) was the most preferred. Gender differences showed a tendency, with women displaying higher preferences for music listening. For educational level, participants with a high school diploma or higher showed a tendency to prefer cognitive videos. 

### 3.2. Design Phase Findings

In the design phase, the structure and function of the user application were configured to be easy to use by elderly individuals with MCI or dementia. The user application consists of the following major parts: cognitive improvement, health care, emotional support, motivation, and caregiver call. First, the cognitive improvement component consisted of cognitive games, cognitive videos, and schedule management. Cognitive games enabled various cognitive stimulations combined with daily life skills through concentration, memory, verbal thinking skills, space, time, and calculation. They were composed using many pictures and colors that made them interesting. A total of 44 games were created, and the level was automatically adjusted according to the level of the elderly individual. Based on the systematic review, the program was designed so that it could be performed three times a week for 30 to 40 min. After completing the essential learning, users could voluntarily conduct additional learning. For the cognitive videos, scenarios were written by a veteran in the field of exercise for the elderly, and the cognitive improvement and muscle exercises were produced by the researchers. After completing the essential learning, elderly individuals could voluntarily watch additional videos. Schedule management made it easy for users to directly input their schedules. The appointment data could be added to the schedule of the users from the administrator server.

Second, the health care component consisted of online classes, health notes, and cognitive improvement nutrition. Online classes allowed the researchers to participate in non-face-to-face classes every week and could be easily accessed through a pop-up notification before the class started. In health notes, information measured through smart devices, such as blood pressure, pulse, oxygen saturation, blood sugar, steps, and weight, could be viewed. This information was graphically represented, with abnormal values displayed in separate colors so that elderly individuals could easily recognize and understand them. Cognitive improvement nutrition enabled them to record the daily intake of seven nutrients, along with educational video materials describing a good diet. The application set an alarm at a designated time to help users record data every day.

Third, the emotional support component consisted of video calls, listening to music, and the community. The video call consisted of a list of people desired by users, including one researcher. Users could try to make a call by simply pressing a photo without looking for a separate phone number. For listening to music, a player was newly developed so that the elderly individuals could intuitively use it. The music comprised Korean traditional songs (Trot), popular songs, religious music, and sounds of nature that seniors like. A social networking service was cyberspace where users could not only communicate with other elderly people with MCI or dementia but also with the researchers, and they could share photos by uploading them to communicate with each other. In addition to commenting on the community, users could also prescribe various emoticons.

Fourth, the motivation component consisted of a daily to-do list and goal achievement. The task was set for each day, and when the achievement criteria were met, it was automatically displayed as completed. There were six tasks for the day, and if users completed five or more of these tasks, they were considered to have completed their tasks. When users performed more than five tasks, they were considered to have achieved the goal and then received a token as a reward. Moreover, users could also check the status of the last two weeks of goal achievement.

Lastly, the caregiver call component had a click button with call, fall, and wandering monitoring functions. The button click call function could be used through a button on the smart necklace worn by users. When the button was pressed, a notification was sent to the two representative caregivers. The fall and wandering detection functions were also detected using the smart necklace.

The structure and function of the administrator were designed for convenient user management. The application comprised two major functions: management and communication. The administrator could manage issues such as whether the daily to-do list had been completed, the status of incentives, monitoring health status, and data downloading. They could check whether a used had participated in the daily to-do list, which was an essential program, and manage incentives according to the number of times the daily to-do list was completed. Furthermore, there was an incentive management page where administrators could see the number of tokens users had received for doing well on the to-do list. When the application was executed, the administrator could check the user’s abnormal signs, wandering, and caregiver calls on the main board. Additionally, administrators could view the list of users, and when they clicked on the desired user, they could see the information and execution status. Every participant’s performance status could be viewed graphically with recorded information such as vital signs, nutritional intake, and cognitive self-diagnosis. There was a menu where the administrator could download the information measured by the user using a smart device and the tablet use data as an Excel file. Also, the administrator was able to communicate with users with video calls and community. 

### 3.3. Development Phase Findings

**Figure 3:**
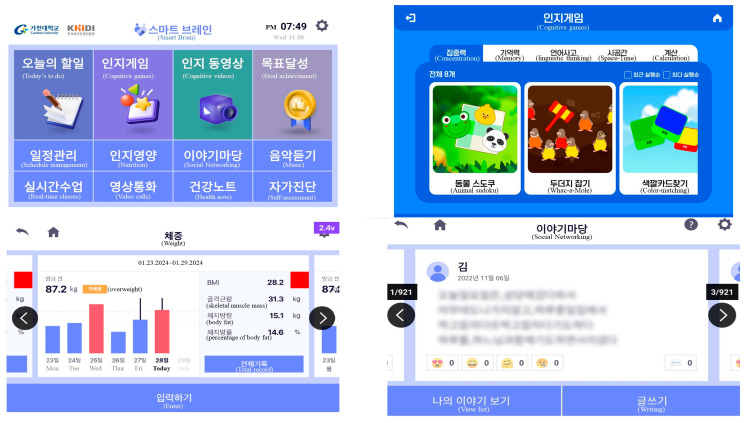
Screen of Smart Brain.

In the development phase, we created the application and named it “Smart Brain” (Figure 3). Based on the Android system, Smart Brain was developed according to the size of the tablet (Lenovo, 10.1 inches, 1920 × 1200).

In the user server, various sounds and images that could appeal to the target audience were inserted. Image materials included in the programs were drawn by the researcher and then edited by the designer, or in some cases, the researcher took pictures or used free images processed by the researcher and designer. Sounds selected were those preferred by the elderly and were available for free. A total of five videos were produced, three related to cognitive improvement exercises and two related to dementia information education. Various sound effects were added when users pressed a button to solve cognitive games or perform missions.

In the administrator server, we could check and download the records of all users. When a user used a blood pressure monitor, blood glucose meter, scale, or smartwatch, data were transmitted to a tablet that acted as a home hub. After interlocking the tablet, data were transmitted to the administrator server. Furthermore, when a user used an application on a tablet, it was transmitted directly to the administrator server.

### 3.4. Implementation and Evaluation Phase Finding: Usability Test of Uses and Experts

The application was evaluated using the MARS tool with seven experts, including two geriatric experts, two computational experts, one nursing professor, and two nurses. The application was evaluated at > 3 points in all areas, except for wandering monitoring. The mean score of the quality of the application was 4.00 ± 0.19. In descending order, the scores in each category were app subjective quality (4.29 ± 0.39), functionality (4.00 ± 0.41), engagement (3.94 ± 0.46), information (3.92 ± 0.25), and esthetics (3.86 ± 0.26) (Table 3).

Furthermore, uMARS was evaluated after using the application with 11 elderly people with MCI or dementia attending daycare centers (Table 4). The application was also evaluated higher than 3 points, except for a willingness to pay for the app. The average score for the overall application quality evaluation was 3.80 ± 0.24. The scores in each category, ranked from highest to lowest, were information (4.16 ± 0.42), esthetics (4.12 ± 0.43), engagement (3.78 ± 0.63), functionality (3.78 ± 0.63), and app subjective quality (3.66 ± 0.49).

Based on feedback from the participants, it was noted that some users were unfamiliar with tablet touch interfaces, resulting in difficulty with accurate touch operations. Additionally, the time limits for cognitive games were reported to be either too fast or too slow for some users. To address these issues, we plan to introduce a touch pen to improve usability and adjust the cognitive game time limits in future updates.

## 4. Discussion

This study developed a cognitive training program, Smart Brain, using ICT-based on a systematic review and needs and preference analysis to provide comprehensive interventions for community-dwelling older adults with MCI or dementia. Smart Brain has significant potential as a tool to address early cognitive decline and delay its progression. While the current study focuses on individuals with early signs of cognitive impairment, future applications could target populations with normal cognitive function but at increased risk of cognitive decline, enhancing its preventive potential. Early implementation in public health programs may proactively address cognitive decline, potentially delaying the onset of dementia. The first strength of Smart Brain is that it motivates and provides feedback to users to continue using the application. Second, the application is a comprehensive program that not only promotes cognition but also combines the ability to perform daily activities. Third, users can maintain social interaction through our application. Finally, the usability was increased to make it easier for the elderly to use.

The advantage of our application is that it is optimized for the motivation of elderly individuals with MCI or dementia. FindMyApps [30] recommends encouraging users to engage with an application at least a few times a week to increase engagement. In contrast, in Smart Brain, tasks are written on daily to-do lists, and a token is given when the mission is completed. In particular, participants in this study had a higher preference for motivation in the development of digital care devices. The researchers designed Smart Brain by reflecting on the analysis. Therefore, unlike the eMIND [31], Smart Brain has a variety of ways to motivate users. In the elderly with dementia, motivation is reduced not only because of cognitive decline but also because of depressive symptoms and apathy [32]. Therefore, Smart Brain was designed to motivate users while they used it. Incentives are provided according to the accumulated number of stamps, and users can check the number of stamps they have received per month. Additionally, administrators can provide feedback through video calls and pop-up notifications.

Smart Brain is a comprehensive cognitive training program that adds daily life skills to general cognitive training. The inability to perform activities of daily living (ADL) is a key symptom of dementia and a prerequisite for diagnosis [33]. ADL is closely related to cognitive function [34] and affects the caregivers’ burden [35]. However, most cognitive games are not closely related to everyday life [36,37]. In contrast, Smart Brain’s cognitive game uses real photos that can express daily life well. For example, the cooking sequencing game explains the order in which dishes are frequently eaten based on real photos. Furthermore, problems encountered in daily life can be solved through games in this application. Therefore, these cognitive games are considered close to daily life, and not only do they contribute to cognitive improvement in people with dementia but ADL improvement can also be expected.

Another strength of Smart Brain is that it allows users to maintain social interactions with their family, friends, and administrators. For elderly individuals with dementia, social interaction has been proven to have positive effects, such as preventing cognitive decline [38] and reducing the behavioral and psychological symptoms of dementia [39]. In this study, the desire of healthy elderly people to communicate with others in a community as well as the researcher reflected their need for an application like this. Unlike other cognitive training programs [36], elderly individuals with MCI or dementia can use this application’s video calls to talk to friends, family, and administrators at any time. Furthermore, users can communicate with many elderly dementia patients using the social networking service that functions without reminding nearby friends and family of social support [40]. Functions such as video calls and social networking services that maintain social interaction in Smart Brain will help improve the symptoms of MCI or dementia in the elderly. These benefits of using ICT for intervention contributed to the strong belief that it could improve the quality of life for elderly with dementia and/or caregivers.

Finally, the Smart Brain application is easy to use for the elderly with MCI or dementia. According to the needs and preference survey in this study, the biggest reason for not wanting to use a non-face-to-face service was that it would be difficult to use a new device. Therefore, we designed a device using large letters, pictograms, and striking colors for convenient use, especially for those who are not very familiar with devices. Additionally, users receive a pop-up notification for tasks that must be performed at a specific time, and if they click the notification, they are automatically redirected to the corresponding page. Thus, the design and interface of the application will improve the usability and quality of experience. These design considerations were confirmed by the usability evaluation. Experts rated the application highly for subjective quality (4.29 ± 0.39) and functionality (4.00 ± 0.41), while users with MCI or dementia provided high scores for information (4.16 ± 0.42) and esthetics (4.12 ± 0.43). This alignment between design features and evaluation outcomes suggests that the program effectively addresses accessibility challenges faced by older adults, contributing to an improved quality of experience. However, one possible reason for the relatively low score in the evidence-based component (2.71 ± 0.49) is the limited visibility of how systematic review findings were integrated into the development of the application. Although the application was designed using evidence from the systematic review, this connection may not have been explicitly communicated or sufficiently detailed in the content. Future updates will aim to address this by incorporating additional evidence, providing transparent data integration, and explicitly linking features to specific evidence-based practices.

A limitation of the Smart Brain application is that it is currently tailored for Android devices due to time and resource constraints. Furthermore, elderly people with MCI or dementia reported that they had a shorter time limit for reading and solving problems in cognitive games. Based on the feedback, we plan to adjust the time appropriately by considering them as our target audience. In the functionality part, there were problems caused by overlapping touches because elderly people were not familiar with using a tablet, and a touch pen will be provided in the future. Another limitation is the small sample size of the usability testing, which may constrain the generalizability of the findings. Moreover, the study was limited by the short evaluation period and the lack of detailed participant characteristics, such as age, gender, or socioeconomic background, which could influence the findings. These variables could significantly influence the effectiveness and usability of Smart Brain, and future studies will explore their impact to optimize the application for diverse populations. While the feedback provided valuable insights for improving the application, future studies will involve larger and more diverse samples to validate the effectiveness and usability of the program across broader populations. Finally, although the participants gave a good overall evaluation of the program, they reported that they would not pay to use it. This low willingness to pay could be attributed to economic constraints, the perceived value of the application, or competition from free alternatives. To address this issue, hybrid revenue models could be considered, such as offering basic features for free while charging for premium functionalities. We suggest that this application should be supported by the government so it can be actively used in public health and daycare centers for elderly individuals with MCI or dementia.

## 5. Conclusions

This study developed a comprehensive cognitive training program, Smart Brain, using ICT for elderly individuals with MCI or dementia. The program design was informed by a systematic review, needs assessment, and preference analysis. Smart Brain includes cognitive games, physical activity, emotional support, health care, motivation, and call sign, with particularly specialized components in motivation and social interaction compared to other applications. The usability of the application has been improved for the target audience and based on the usability test results, Smart Brain can be made easier to use. Moreover, administrators can efficiently manage many elderly people with MCI or dementia without restrictions on time and place. It is important to note, however, that the current findings are based on preliminary data from a limited sample size, which may not be representative of all patients with cognitive disorders. Further studies with larger and more diverse participant groups are needed to validate these findings and assess the program’s effectiveness comprehensively. Despite these limitations, the development of Smart Brain represents a promising step forward and holds important clinical significance for nurses managing community-dwelling elderly individuals with MCI or dementia.

## Figures and Tables

**Table 1:** Baseline characteristics of older adults in the needs and preference survey (n = 100).

Variable	Contents	Mean ± SD ^1^/N (%)
Age		73.2 ± 7.0
Gender	Male	33 (33.0%)
	Female	67 (67.0%)
Subjective economic status	High	13 (13.0%)
	Moderate	67 (67.0%)
	Low	20 (20.0%)
Education level	No formal education	7 (7.0%)
	Elementary/Middle school	31 (31.0%)
	High School/college	60 (60.0%)
	Graduate or Higher	2 (2.0%)
Primary caregiver	Spouse	54 (54.0%)
	Child	22 (22.0%)
	Sibling	1 (1.0%)
	Caregiver	1 (1.0%)
	None	22 (22.0%)

^1^ SD, standard deviation.

**Table 2:** Preferred contents of cognitive training program using ICT for spouses of people with dementia (n = 100).

Category	Contents	Mean ± SD ^1^
Cognitive improvement	Cognitive games	3.68 ± 1.11
	Cognitive videos	3.26 ± 1.27
	Schedule management	3.05 ± 1.14
Health care	Online classes	3.06 ± 1.32
	Health note	3.67 ± 1.17
	Cognitive improvement nutrition	2.78 ± 1.13
Emotional support	Video calls	3.20 ± 1.19
	Listening to music	3.37 ± 1.15
	Social networking service	3.41 ± 1.25
Motivation	Today’s to-do list	3.48 ± 1.21
	Goal achievement	3.49 ± 1.29
Caregiver call	Button click call	3.38 ± 1.30
	Fall monitoring	3.09 ± 1.24
	Wandering monitoring	2.99 ± 1.32

^1^ SD, standard deviation.

**Table 3:** Usability assessment of experts (n = 7).

Category	Component	Score (Mean ± SD ^1^)
Quality of application	Mean score	4.00 ± 0.19
Engagement	Mean score	3.94 ± 0.36
	Entertainment	3.71 ± 0.76
	Interest	4.00 ± 0.58
	Customization	4.14 ± 0.90
	Interactivity	4.00 ± 0.82
	Target group	3.86 ± 1.07
Functionality	Mean score	4.00 ± 0.41
	Performance	4.00 ± 0.81
	Ease of use	3.71 ± 0.76
	Navigation	4.14 ± 0.69
	Gestural design	4.14 ± 0.69
Esthetics	Mean score	3.86 ± 0.26
	Layout	3.86 ± 0.90
	Graphics	3.71 ± 1.11
	Visual appeal	4.00 ± 0.82
Information	Mean score	3.92 ± 0.25
	Accuracy of app description (in app store)	4.14 ± 0.69
	Goals	4.29 ± 0.76
	Quality of information	4.00 ± 0.82
	Quantity of information	4.29 ± 0.76
	Visual information	4.14 ± 0.90
	Credibility	3.86 ± 0.90
	Evidence base	2.71 ± 0.49
App subjective quality	Mean score	4.29 ± 0.39
	Willingness to recommend app to others	4.71 ± 0.49
	Estimated number of uses per year	3.86 ± 0.90
	Willingness to pay for app	4.43 ± 0.98
	Overall star rating of app	4.14 ± 1.07

^1^ SD, standard deviation.

**Table 4:** Usability assessment of participants with mild cognitive impairment or mild dementia (n = 11).

Category	Component	Score (Mean ± SD ^1^)
Quality of application	Mean score	3.80 ± 0.24
Engagement	Mean score	3.78 ± 0.63
	Entertainment	3.64 ± 1.12
	Interest	3.73 ± 1.01
	Customization	4.00 ± 1.00
	Interactivity	3.82 ± 0.98
	Target group	3.73 ± 1.01
Functionality	Mean score	3.78 ± 0.63
	Performance	3.09 ± 0.83
	Ease of use	3.55 ± 0.93
	Navigation	3.27 ± 0.79
	Gestural design	3.27 ± 0.79
Esthetics	Mean score	4.12 ± 0.43
	Layout	3.09 ± 0.83
	Graphics	4.18 ± 0.87
	Visual appeal	4.09 ± 1.04
Information	Mean score	4.16 ± 0.42
	Quality of information	4.09 ± 0.94
	Quantity of information	4.09 ± 0.94
	Visual information	4.18 ± 0.87
	Credibility	4.27 ± 0.91
App subjective quality	Mean score	3.66 ± 0.49
	Willingness to recommend app to others	4.64 ± 0.51
	Estimated number of uses per year	4.00 ± 0.89
	Willingness to pay for app	2.27 ± 1.01
	Overall star rating of app	3.73 ± 1.61

^1^ SD, standard deviation.

## Data Availability

The data presented in this study are available on request from the corresponding author.
